# CTI-2 Inhibits Metastasis and Epithelial-Mesenchymal Transition of Breast Cancer Cells by Modulating MAPK Signaling Pathway

**DOI:** 10.3390/ijms222212229

**Published:** 2021-11-12

**Authors:** Junfeng Ke, Wenzhao Han, Fanwei Meng, Feng Guo, Yuhong Wang, Liping Wang

**Affiliations:** 1 Key Laboratory for Molecular Enzymology and Engineering, Ministry of Education, Jilin University, Changchun 130012, China; kejf19@mails.jlu.edu.cn (J.K.); hanwz18@mails.jlu.edu.cn (W.H.); Mengfw19@mails.jlu.edu.cn (F.M.); fengguo19@mails.jlu.edu.cn (F.G.); chao_lake@gmail.com (Y.W.); 2 School of Life Sciences, Jilin University, Changchun 130012, China; 3 Engineering Laboratory for AIDS Vaccine, Jilin University, Changchun 130012, China

**Keywords:** breast cancer, metastasis, EMT, MAPK

## Abstract

Although some breast cancer patients die due to tumor metastasis rather than from the primary tumor, the molecular mechanism of metastasis remains unclear. Therefore, it is necessary to inhibit breast cancer metastasis during cancer treatment. In this case, after designing and synthesizing CTI-2, we found that CTI-2 treatment significantly reduced breast cancer cell metastasis in vivo and in vitro. Notably, with the treatment of CTI-2 in breast cancer cells, the expression level of E-cadherin increased, while the expression level of N-cadherin and vimentin decreased. In addition, after CTI-2 treatment, those outflow levels for p-ERK, p-p38, and p-JNK diminished, while no significant changes in the expression levels of ERK, JNK, or p38 were observed. Our conclusion suggested that CTI-2 inhibits the epithelial-mesenchymal transition (EMT) of breast carcinoma cells by inhibiting the activation of the mitogen-activated protein kinase (MAPK) signaling pathway, thereby inhibiting the metastasis of breast tumor cells. Therefore, we believe that CTI-2 is another candidate for breast tumor medication.

## 1. Introduction

Cancer has become a major cause of mortality globally. Breast cancer plays a pivotal role in causing cancer deaths in women [1,2]. Breast cancer accounts for a significant proportion of the newly diagnosed cancer cases in American women [3], and its incidence and mortality rates continue to increase every year [4,5,6]. Although early detection improves clinical outcomes, metastasis to distant organs still leads to a high mortality rate in breast cancer patients [7]. Thus, it has been established that distal metastasis is a principal reason for death in breast tumor patients [8].

EMT is considered to be a salient process of tumor migration and invasion in patients with many cancers [9,10], inclusive of breast cancer [11]. The key features of EMT include the decrease in adhesion between cells or cells and the matrix, and cellular polarity, which leads to migration and invasion [12,13,14]. During the EMT process, epithelial cells undergo significant cytoskeletal rearrangement and transform into a mesenchymal phenotype with a fusiform cell shape and an increased ability for migration and invasion [15,16,17]. In addition, the expression of E-cadherin in cancer cells decreases [18], while the expression of N-cadherin and vimentin increases [19,20,21,22]. Therefore, the activation of EMT is considered a key process for the development of tumor metastasis [23,24,25].

MAPK signaling pathway was discovered some time ago. The research on this pathway has been extensive. The MAPK pathway is often related to cancer development and is a critical focus for cancer medication [26]. Studies have shown that the MAPK cascade reaction pathway is often activated in the progression of gastrointestinal malignant tumors [27]. The MAPK pathway additionally plays a few critical physiological parts in normal cells. For example, MAPKs manage cell proliferation and apoptosis [28]. The modulation of transcription factors by MAPKs results in gene transcription and response to signals in the cell [29]. Abnormal MAPK signaling has been thought to be related to many malignant tumors, such as breast cancer, lung cancer, glioma, and colorectal cancer. The abnormality regulation of the MAPK signal in malignant tumors is related to cell escape from apoptosis, abnormal proliferation, chemotherapy resistance, and drug resistance [30]. In humans, the MAPK family mainly includes three parts, named the extracellular signal-regulated kinase (ERK), p38, and the Jun N-terminal kinases (JNK), [31,32,33]. The MAPK pathway is a signaling pathway that is very important in promoting the development of breast cancer. It is believed to play an important role in the invasion and metastasis of breast tumor cells [34,35,36].

Here, we modified Metarrestin (ML246, a small molecule tumor metastasis inhibitor that works by promoting the disassembly of the perinucleolar compartment) to obtain a better tumor metastasis inhibitor, and then through screening, we obtained CTI -2, which has a potential inhibition effect on tumor metastasis. The effect on breast tumor cells and the mechanism of action were then explored. Our outcomes indicate that CTI-2 can inhibit breast cancer metastasis, and show that EMT-related genes and the MAPK signaling pathway play important roles in this process.

## 2. Results

### 2.1. CTI-2 Affected the Migration of Breast Carcinoma Cells

**Figure 1:**
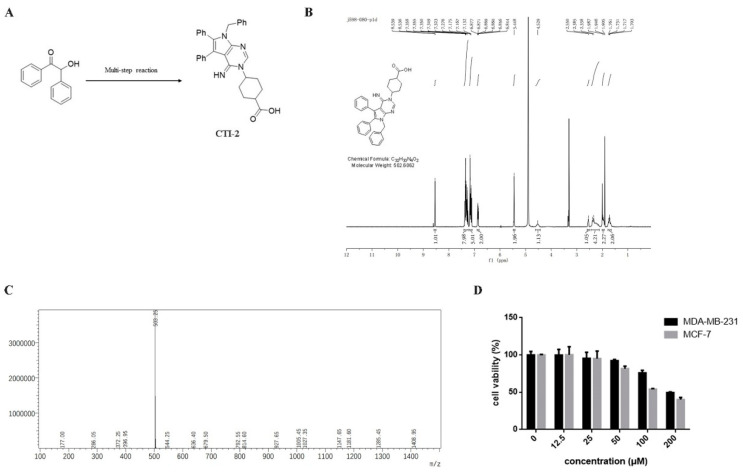
Molecular structure of CTI-2. (**A**) Compound 1 obtains CTI-2 through a multi-step reaction. (**B**) Molecular structure and NMR spectrum of CTI-2. (**C**) Mass spectrometry of CTI-2. (**D**) Cells were processed with CTI-2 for 24 h and the viability was detected by MTT assay. Each value is expressed as the mean ± standard deviation (SD) (*n* = 3).

**Figure 2:**
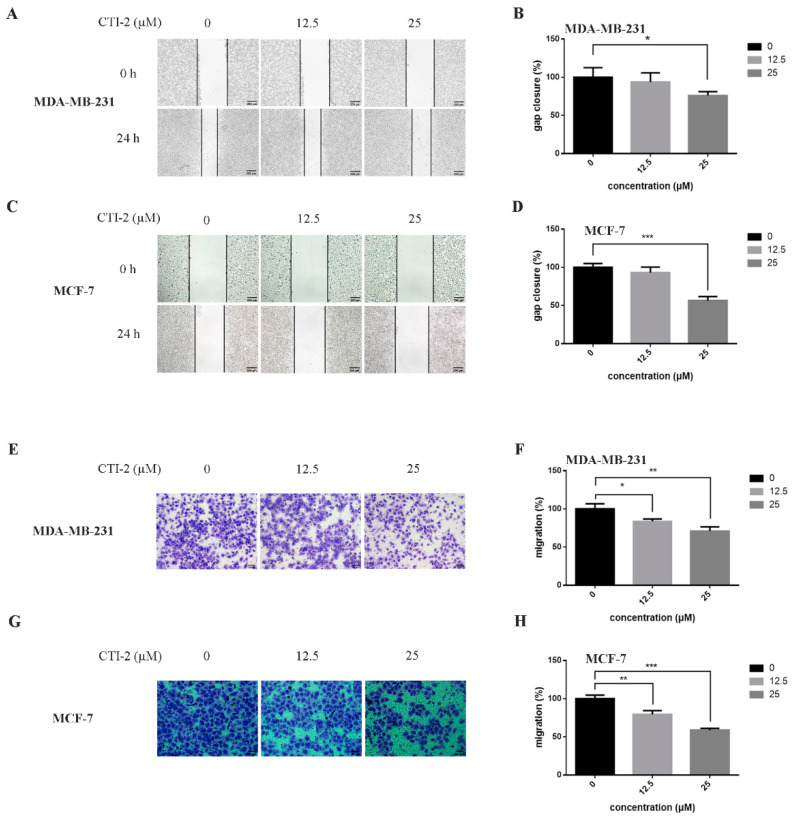
CTI-2 affected the migration of breast carcinoma cells. (**A**,**C**) Images of breast cancer cells incubated with CTI-2 for 24 h scratching were captured (Scale bar: 200 µm). (**B**,**D**) Assess the ability of CTI-2 to inhibit the closure of specified cell gaps through Image J. (**E**–**H**) Breast cancer cells were treated with CTI-2 and Transwell analysis was carried out. After culturing for 24 h, the metastasis cells were imaged and measured (Scale bar: 50 µm). Each value is expressed as the mean ± SD (*n* = 3).

We obtained CTI-2 through a series of synthesis (Figure 1A) and purification (Figure 1B,C). To figure out the impact of CTI-2 on the viability of breast tumor cells, the indicated concentration (0 µM, 12.5 µM, 25 µM, 50 µM, 100 µM, 200 µM) of CTI-2 was added to breast cancer cells followed by culturing for 24 h. The viability of each group of cells was gauged using the MTT assay. At the concentrations of 12.5 µM and 25 µM, CTI-2 did not significantly change the viability of MDA-MB-231 and MCF-7 cells (Figure 1D). We investigated the impact of CTI-2 on breast tumor cell migration using wound healing and transwell assays. Compared with the control group, the migration distance of MDA-MB-231 cells (Figure 2A,B) and MCF-7 cells (Figure 2C,D) was significantly reduced after CTI-2 treatment. Moreover, the transwell migration assay results also demonstrated that CTI-2 can restrict the migration ability of MDA-MB-231cells (Figure 2E,F) and MCF-7 cells (Figure 2G,H). Notably, treatment with 25 μM of CTI-2 resulted in a more prominent inhibitory effect on migration. In addition, we also explored whether CTI-2 can affect the colon cancer cell line HCT-116 (Supplementary Figure S1). Although CTI-2 produced significant effects on both MDA-MB-231 and MCF-7 cell lines, we finally selected the triple-negative breast cancer cell line MDA-MB-231 for follow-up research.

### 2.2. CTI-2 Inhibits EMT in Breast Carcinoma Cells

**Figure 3:**
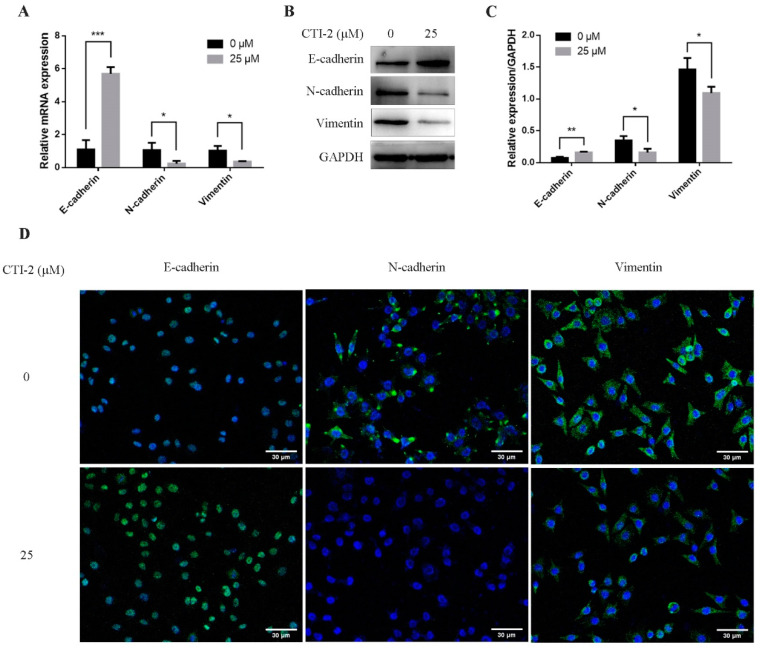
Cells were incubated with or without CTI-2 for 24 h, and the expression of the specified protein in the corresponding group was observed by qRT-PCR (**A**) and western blot (**B**). Statistics on the expression of EMT regulatory proteins (**C**). GAPDH as an internal reference. (**D**) Immunofluorescence staining image of the corresponding group of cells. (Scale bar: 30 µm) Blue indicates the nucleus and green represents the protein. Each value is expressed as the mean ± SD (*n* = 3).

EMT was assumed to be a vital part of breast cancer migration, which is mainly manifested by diminishing E-cadherin expression while enhancing the expression of N-cadherin and vimentin. To explore the potential molecular mechanism of CTI-2 inhibiting metastasis, we performed qRT-PCR and western blotting, to determine whether CTI-2 modulated EMT in breast cancer cells. The level of E-cadherin, a central molecule involved in the assessment of EMT progression, was significantly enhanced after treatment with the specified dose of CTI-2. The increment of the expression levels of other important cadherins, for instance, N-cadherin and vimentin, indicated the progression of the EMT process. Here, CTI-2 significantly diminished the expression of N-cadherin and vimentin at a specific dose (Figure 3A–C). The same results were obtained with representative confocal immunofluorescence staining (Figure 3D). Hence, it was concluded that CTI-2 triggered EMT in breast carcinoma cells.

### 2.3. RNA Sequencing Reveals That Pathways Were enriched in CTI-2 Treated Breast Carcinoma Cells

**Figure 4:**
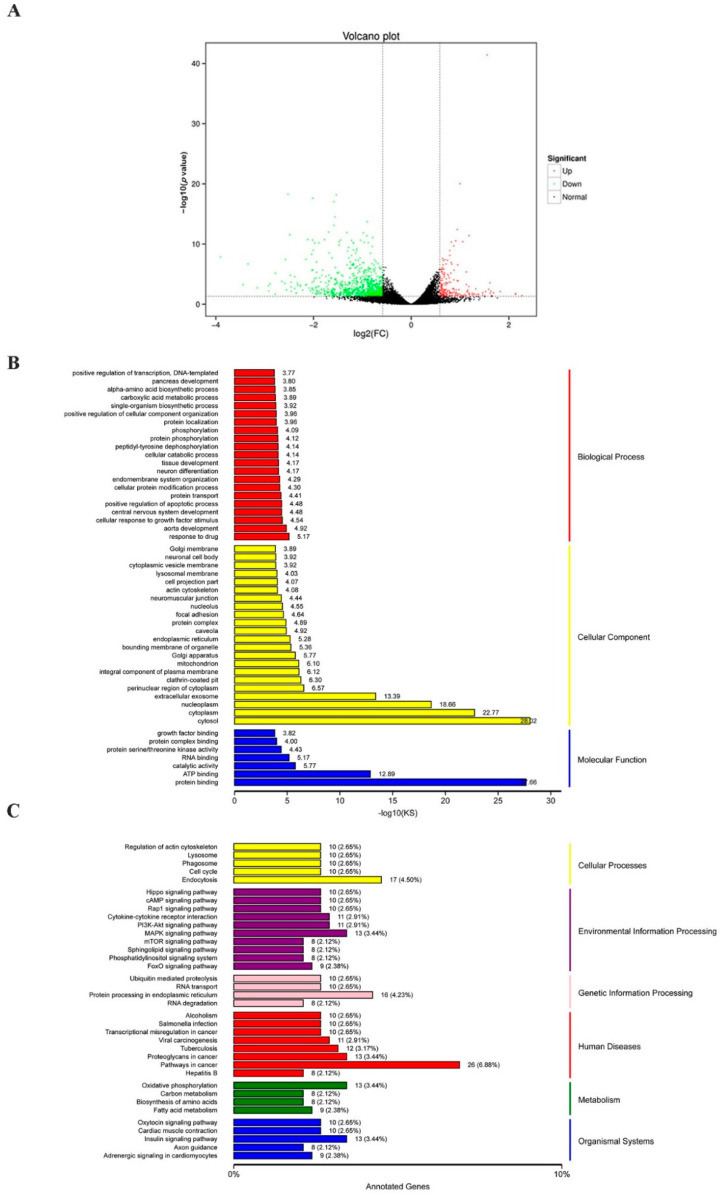
Various pathways were affected by the treatment of CTI-2. (**A**) The volcano plot (*p* < 0.05, fold change ≥1.5). (**B**) GO analysis of genes significantly regulated in CTI-2 treated MDA-MB-231 cells. (**C**) KEGG pathway analysis to determine the main pathways that may be involved in the process.

To determine the possible mechanism by which CTI-2 inhibits breast cancer metastasis, RNA sequencing (RNA-seq) was carried out to evaluate the genetic altering in the control group and the CTI-2 treatment group. The results revealed that 211 and 844 genes were upregulated and downregulated, respectively, in CTI-2-treated breast cancer cells, compared to those in the controls (Figure 4A). Furthermore, gene ontology (GO) analysis results indicated that differentially expressed genes (DEGs) are aggregated by protein binding when molecular functions were performed in response to drugs; this was also observed in the cytosol surrounding cellular components (Figure 4B). Moreover, the Kyoto encyclopedia of genes and genomes (KEGG) pathway analysis was carried out to determine the main pathways that may participate in this process. From that figure, we received the signaling pathways involved in endocytosis, MAPK signaling, protein processing in the endoplasmic reticulum, cancer development, oxidative phosphorylation, insulin as those that were primarily affected (Figure 4C). It’s worth noting that some of them, for example, the MAPK signaling, are generally considered to be an important part of breast cancer development [37]. Therefore, our subsequent experiments investigated whether CTI-2 hindered the metastasis of breast cancer through the MAPK pathway.

### 2.4. CTI-2 Blocks the MAPK Signaling Pathway in Human Breast Carcinoma Cells

**Figure 5:**
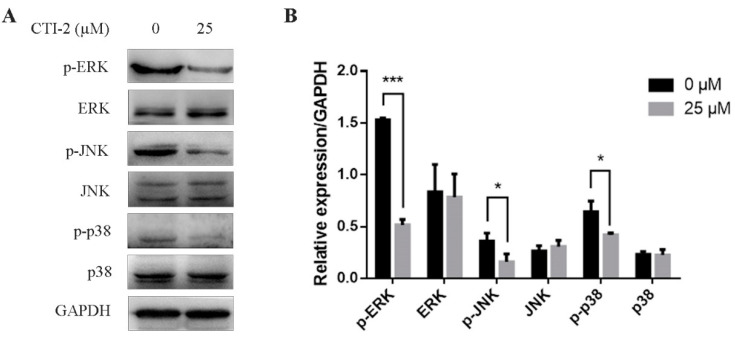
CTI-2 inhibits phosphorylation of MAPK pathway protein in breast tumor cells. Cells were dealt with or without CTI-2 for 24 h (**A**) and the expression of MAPK-related molecules in the corresponding group was analyzed by western blotting. GAPDH as an internal reference. (**B**) Statistical analysis of the expression of the above specified proteins. Each value is expressed as the mean ±SD (*n* = 3).

The MAPK signaling pathway is a well-known pathway in cells. Abnormal activation of the MAPK signaling pathway can promote the EMT process, thereby promoting the migration of breast carcinoma cells. We tested the impact of CTI-2 on the MAPK signaling pathway in human breast carcinoma cells via western blotting. The consequences showed that in MDA-MB-231 cells which were incubated with CTI-2 for 24 h, the expression levels of p-ERK, p-JNK, and p-p38 reduced significantly, while the expressions of ERK, JNK, and p38 did not change significantly (Figure 5A,B). These results suggested that CTI-2 could restrain the MAPK signaling pathway in human breast tumor cells.

### 2.5. CTI-2 Impeded the Metastasis of Human Breast Carcinoma Cells in vivo

**Figure 6:**
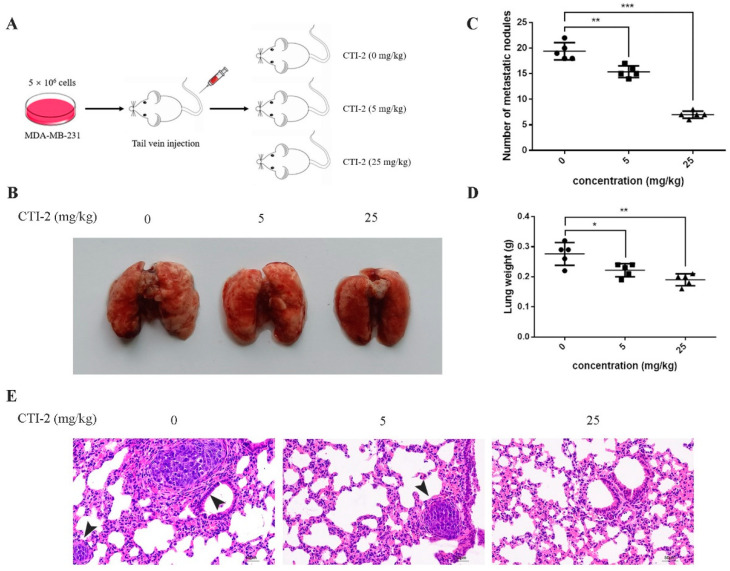
CTI-2 impeded the metastasis of human breast carcinoma cells in vivo. (**A**) Schematic demonstrating the metastasis assay model in vivo. (**B**) After 3 weeks of injecting breast cancer cells (MDA-MB-231) through the tail vein, there were macroscopically visible lung metastatic nodules in BALB/c mice. (**C**) The number of metastatic nodules. (**D**) Statistical analysis of the weight of the lungs of each group of mice. (**E**) Representative images of H&E staining for histological analysis of lung (×200 magnification) (Scale bar: 50 µm). Black arrows indicate larger metastatic lesions. Scale bar, 50 µm. Each value is expressed as the mean ± SD (*n* = 5).

We injected MDA-MB-231 cells into the tail vein of 15 female BALB/c mice to establish tumor metastasis models. Then, we classified the mice into three groups (five mice in each group) after one week. Mice in each group received CTI-2 (0 mg/kg, 5 mg/kg, 25 mg/kg) intraperitoneally once every two days (Figure 6A). The corresponding mice were sacrificed after two weeks of constant medication. The lungs of all mice were carefully removed and flushed with saline. The metastatic nodules were then observed with the naked eye. Subsequently, tissue fixative was used to preserve the lungs for further H&E staining. The lungs of mice without CTI-2 had multiple metastatic nodules and metastatic lesions, while the lungs of mice with CTI-2 (5 mg/kg, 25 mg/kg) had fewer metastatic nodules and metastatic lesions. (Figure 6B). The total number of metastatic nodules and lung weight in each group was measured (Figure 6C,D). We carried out H&E staining and microscopically examined the lung tissues of mice belonging to different groups under an inverted microscope (200× magnification). We found that the lungs of the untreated mouse group showed more metastatic nodules and metastatic lesions compared to the CTI-2 treated mouse group (Figure 6E).

## 3. Discussion

Breast tumors are among the most common malignant tumors and account for a large proportion of female cancers worldwide. The survival rate of patients with early breast cancer is relatively high, but that of patients with advanced breast cancer who have undergone distant metastasis is only about 11%. Triple-negative breast cancer (TNBC) is a specific subtype of breast cancer that does not express an estrogen receptor (ER), a progesterone receptor (PR), or human epidermal growth factor receptor 2 (HER-2). Its clinical features include high invasiveness, high metastatic potential, easy relapse, and poor prognosis. In addition, there is a large amount of heterogeneity in the triple-negative subtype, which makes finding a common target a challenge. At the same time, TNBC tumors lack the expression of ER, PR, and HER2. It is also not sensitive to endocrine therapy or HER2 therapy, so the main methods currently used for triple-negative breast cancer treatment include surgery, chemotherapy, and radiation therapy [38]. However, patients with distant metastases are likely to experience recurrence after surgery; hence, overcoming breast cancer metastasis is the key to treatment. In this case, we found that CTI-2 may inhibit the metastasis of human breast tumors such as MDA-MB-231 by using wound healing and transwell assays. Therefore, we believe that CTI-2 is a potential candidate that could prevent breast cancer metastasis.

During the EMT process, epithelial cells undergo strong morphological changes and transform into mesenchymal cells. EMT has been proven to be an important cause of the separation of cancer cells from the primary tumor, and their metastasis and proliferation to distant regions. Accumulating evidence indicates that EMT plays a key role in carcinoma metastasis and migration, which is positively correlated with an increase in migration. Previous studies have shown that epithelial marker (E-cadherin) and mesenchymal markers (N-cadherin, vimentin) are crucial in tumor cell migration. Herein, our results show that CTI-2 can up-regulate the E-cadherin expression level and significantly down-regulate the expression levels of N-cadherin and vimentin. Our results prove that CTI-2 can restrain the migration of breast tumor cells by inhibiting the EMT process.

Cancer metastasis is mediated by several pathways, including the MAPK pathway. Some studies have indicated that the MAPK pathway is a pathway recognized as regulating tumor metastasis. It is activated frequently and regulates metastasis in human cancers. Our results showed that after MDA-MB-231 cells were incubated with CTI-2, p-ERK, p-p38, and p-JNK levels were down-regulated, while the ERK, JNK, and p38 levels did not change significantly. This could be attributed to the repression of metastasis by CTI-2 via the inhibition of the MAPK signaling pathway.

Finally, we evaluated the potential of CTI-2 to inhibit breast cancer cell metastasis in BALB/c nude mice. Our results indicated that CTI-2 significantly inhibited the metastasis of breast tumor cells into the lungs. The number of metastatic lesions on the lung surfaces of CTI-2-treated mice was significantly reduced in comparison to those untreated mice. The decrease in the number of metastatic nodules in the mouse lung was confirmed by H&E staining. The antimetastatic potential of CTI-2 was found to be significant. In conclusion, CTI-2 indicates the capacity to restrain the movement of breast tumor cells by regulating the MAPK pathway to inhibit the EMT process, which in turn results in the inhibition of breast cancer cell metastasis. It is important to note that while CTI-2 appeared to demonstrate its antimetastatic actions by inhibiting MAPK signaling, there are a number of agents that confer their anticancer actions by activating MAPK signaling. Therefore, the modulation of MAPK to blunt metastases is truly context-dependent. Moreover, because current single-drug therapy makes it easy to produce tumor cells that are drug resistant, which leads to a poor prognosis, most current studies use combination drugs to avoid this situation. Therefore, we speculate that using CTI-2 as a new type of breast cancer inhibitor, when combined with other therapies, may show a better therapeutic effect.

In short, in the follow-up work, we will further optimize CTI-2, and conduct experimental explorations for existing problems, as well as striving to make CTI-2 suitable for most tumor cell lines.

## 4. Materials and Methods

### 4.1. Reagents and Antibodies

The following items were purchased from the indicated sources: BCA protein assay kit was purchased from Dalian Meilun Biotechnology (Dalian, China). primary antibodies against E-cadherin (rabbit, #40860, Signalway Antibody, MD, USA), N-cadherin (rabbit, A5598, Bimake, Houston, TX, USA), Vimentin (rabbit, A5862, Bimake, Houston, TX, USA), phospho-ERK1+2 (rabbit, bs-1522R, Bioss, China), phospho-JNK1+2+3 (rabbit, bs-1640R, Bioss, Beijing, China), phospho-p38 MAPK (rabbit, bs-0636R, Bioss, Beijing, China), ERK1+ERK2 (rabbit, bs-2637R, Bioss, Beijing, China), JNK1+JNK2+JNK3 (rabbit, bs-2592R, Bioss, Beijing, China), p38 MAPK (rabbit, bs-0637R, Bioss, Beijing, China), GAPDH (rabbit, AP0066, Bioworld, MN, USA), Goat Anti-Rabbit IgG(H+L)/HRP (rabbit, bs-40295G-HRP, Bioss, Beijing, China) and Goat Anti Rabbit IgG (H&L)-Alexa Fluor 488 (rabbit, ImmunoWay, Plano, TX, USA).

### 4.2. Cell Line and Cell Culture

MDA-MB-231 and MCF-7 are two breast carcinoma cell lines with strong metastasis and invasion ability, was derived from preserved cells in the laboratory. Dulbecco’s Modified Eagle Medium (DMEM, Meilunbio, Dalian, China) with 10% fetal bovine serum (FBS, Kang Yuan Biology, Tianjin, China) was added to the cells, and then placed in a cell incubator (5% CO_2_, 37 °C).

### 4.3. MTT Assay

Assess the impact of CTI-2 on cell viability via MTT assay. Inoculate MDA-MB-231 and MCF-7 cells (5 × 10^3^ cells/well) in a 96-well plate and culture overnight, and then use different concentrations of CTI-2 processing for 24 h. Then measure cell viability by MTT Cell Proliferation and Cytotoxicity Assay Kit (Solarbio Life Sciences, Beijing, China).

### 4.4. Wound Healing Assay

Inoculate the selected cells (5 × 10^5^ cells/well) in 6-well plates and culture in the incubator until a monolayer was formed. Then, use the tip of a 200 µL pipette to scribble across the cell layer to form regular traces. Then add different concentrations of CTI-2 (0 µM, 12.5 µM, 25 µM) for 24 h and the extent of wound closure was monitored using a fluorescence microscope (Olympus Corporation, Tokyo, Japan).

### 4.5. Transwell Assay

Inoculate the cells (5 × 10^4^ cells/well) in the uppermost chambers of 24-well Transwell plates (Corning, NY, USA), which were full of FBS-free medium, while the lowermost cavities were filled with a medium containing 20% FBS. Specified concentrations of CTI-2 were added to the cell culture medium. After 24 h of incubation, wipe off the cells on the surface of the upper chamber filter, Cells in the lower chamber are fixed with cell fixative and stained with crystal violet. Then use a fluorescence microscope (Olympus Corporation) to take pictures for subsequent counting.

### 4.6. Quantitative RT-PCR

RNA was extracted from different groups through TriQuick (Solarbio Life Sciences, Beijing, China), and RT Master Mix (ABM, Vancouver, BC, Canada) was used for reverse transcription. qRT-PCR was carried using the SYBR^®^ PCR kit (Bimake, Houston, TX, USA). qRT-PCR was performed with the primer pairs of Table S1.

### 4.7. Western Blotting

After being cultured for 24 h in 6-well plates, cells were subsequently incubated with specific concentrations (0 µM, 25 µM) of CTI-2 for 24 h. Subsequently, their total protein content was determined after protein extraction using RIPA lysis buffer (Solarbio Life Sciences, Beijing, China). Use the BCA protein quantification kit to quantify the protein concentration. Then perform SDS-PAGE gel electrophoresis and transfer with 0.2 μm PVDF membrane (Merck, Darmstadt, Germany). After performing the blocking process in 5% skimmed milk (BD, Franklin Lakes, NJ, USA) for 1 h, the primary antibodies and membranes were incubated overnight at 4 °C. Next, discard the primary antibody and cleanse the membrane 3 times with TBST, and then incubated with the secondary antibodies for 1 h at room temperature (RT). Afterward, discard the secondary antibodies and wash the membrane four times in TBST for 10 min. Visualize the resultant antigen-antibody complexes using super sensitive ECL luminescence reagent (Meilunbio, Dalian, China). Images were obtained using the Tanon 5200 (Tanon Science & Technology Co., Ltd., Shanghai, China) imaging system. To normalize the protein load, and GAPDH is regarded as an internal control. Original western blotting images are shown in Supplementary Figures S2–S10.

### 4.8. Immunofluorescence

First, the cells of different groups were fixed with cell fixation solution, and after blocking with 5% bovine serum albumin (BSA). The residual solution was discarded followed by the addition and incubation of the primary antibody at RT for 1 h. Subsequently, cells were incubated with fluorescence-labeled secondary antibodies for 1 h at RT. DAPI (Solarbio Life Sciences, Beijing, China) was used to color cell nuclei. Using the LSM-710 (ZEISS, Germany) inverted fluorescence microscope to take fluorescence images.

### 4.9. RNA-Sequencing

MDA-MB-231 cells were seeded into 60 mm petri dish for 24 h and then incubated with indicated concentrations (0 µM, 25 µM) of CTI-2 for 24 h. Extract the RNA just like before, then use the cDNA-PCR sequencing kit (SQK-PCS109) provided by Oxford Nanopore Technologies (ONT) to prepare 1 ug total RNA for cDNA library. In short, The template conversion activity of reverse transcriptase enriches the full-length cDNA, and directly adds a well-defined PCR linker to both ends of the first strand cDNA. Then use the LongAmp Tag (NEB) to perform 14-loop cDNA PCR. Then using T4 DNA Ligase (NEB) to ligate the PCR product with the ONT adapter. According to the ONT protocol, Agencourt XP beads are used for DNA purification. The final cDNA library was added to the FLO-MIN109 flow cell and run on the PromethION platform of Biomarker Technology Company (Beijing, China). GEO number: GSE188144.

### 4.10. In vivo Metastasis Assay

The procedures for creature examinations were affirmed by the Animal Ethics Committee of Jilin University (Permit Number: 2021SY0505). After acclimatization for a week, MDA-MB-231 (5 × 10^6^) cells were injected into the tail vein of each four to six week old BALB/c female nude mouse (Beijing Vital River Laboratory Animal Technology). One week later, all mice were randomly separated into three groups. Each group received CTI-2 (0 mg/kg, 5 mg/kg, 25 mg/kg) once every two days via the intraperitoneal route. After continuous CTI-2 administration for two weeks, all mice were sacrificed. The lungs of each group were carefully taken out and weighed, and visually count metastatic pulmonary nodules. The lungs of all mice were washed with normal saline and immediately immersed into a Tissue Fixative Reagent (Meilunbio, Dalian, China) for preservation and further H&E staining.

### 4.11. Statistical Analysis

Using GraphPad Prism 6.1 software to execute statistics and analysis on the obtained data. The obtained data are quantitatively expressed as mean ± SD value (*** *p* < 0.001, ** *p* < 0.01, * *p* < 0.05). Statistical comparisons between two groups were performed using the unpaired two-tailed *t*-test. In all results, if *p* < 0.05, the value is considered to be statistically significant.

## 5. Conclusions

The results of our in vitro experiments prove that CTI-2 can restrain the migration of breast carcinoma cells and cause inversion of the EMT process. The inhibition of EMT might occur through the suppression of the MAPK pathway. In addition, the results of the in vivo experiments suggest that CTI-2 inhibits breast cancer cell metastases to the lungs. Therefore, CTI-2 could potentially act as a therapeutic agent against breast cancer.

## Data Availability

Not applicable.

## Supplementary Materials

The following are available online at https://www.mdpi.com/article/10.3390/ijms222212229/s1.
