# Supplementary material for: CTI-2 Inhibits Metastasis and Epithelial-Mesenchymal Transition of Breast Cancer Cells by Modulating MAPK Signaling Pathway
Source: Int J Mol Sci. 2021 Nov 12;22(22):12229. doi: 10.3390/ijms222212229 (PMC8622910; doi:10.3390/ijms222212229)
Supplement: Supplementary file 1 [file ijms-22-12229-s001.zip › ijms-1412350-supplementary.pdf]

Suppl. Table S1. Sequences of primers used for real-time quantitative PCR amplification

Gene		Sequence
E-cadherin	FWD	CAACGACCCAACCCAAGAA
	REV	CCGAAGAAACAGCAAGAGCA
N-cadherin	FWD	TCAGGCGTCTGTAGAGGCTT
	REV	ATGCACATCCTTCGATAAGACTG
Vimentin	FWD	GAACGCCAGATGCGTGAAATG
	REV	CCAGAGGGAGTGAATCCAGATTA

HCT-116

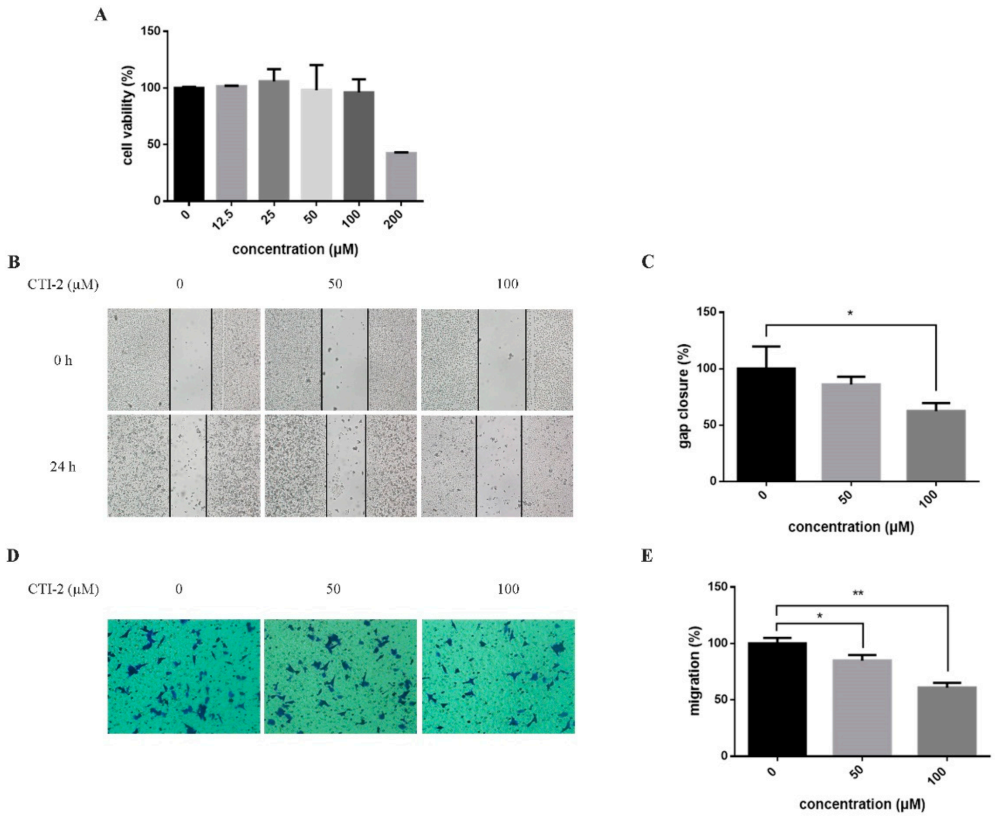

Suppl. Figure S1. CTI-2 affected the migration of HCT-116 cells. (A) Cells were processed with CTI-2 for 24 hours and the viability was detected by MTT assay. (B) Images of HCT-116 cells incubated with CTI-2 for 24 h scratching were captured. (C) Assess the ability of CTI-2 to inhibit the closure of specified cell gaps through Image

J. (D, E) HCT-116 cells were treated with CTI-2 and Transwell analysis was carried out. After culturing for 24 h, the metastasis cells were photo imaged and measured. Each value is expressed as the mean ±SD (n = 3).

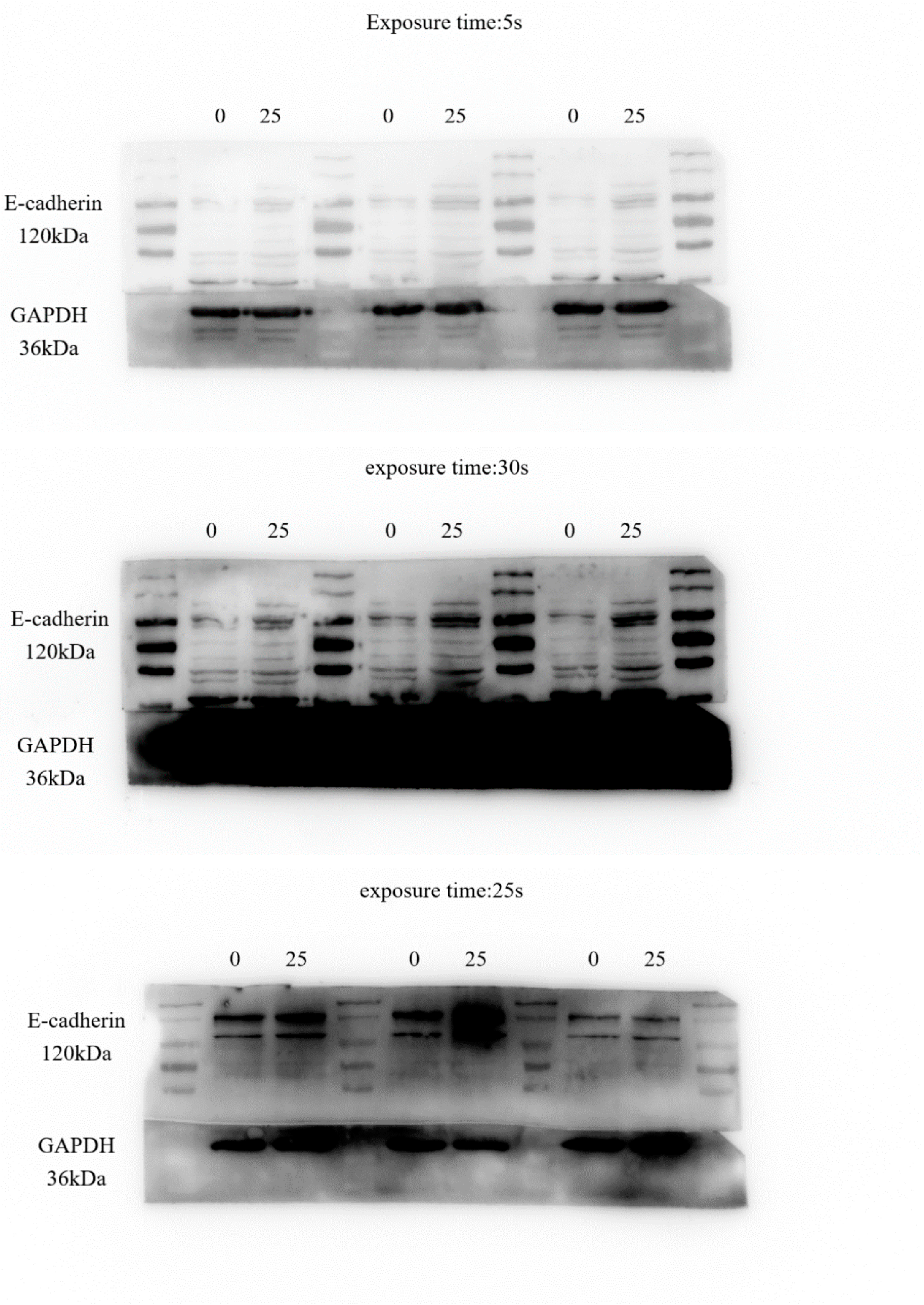

Suppl. Figure S2. Protein expression of E-cadherin.

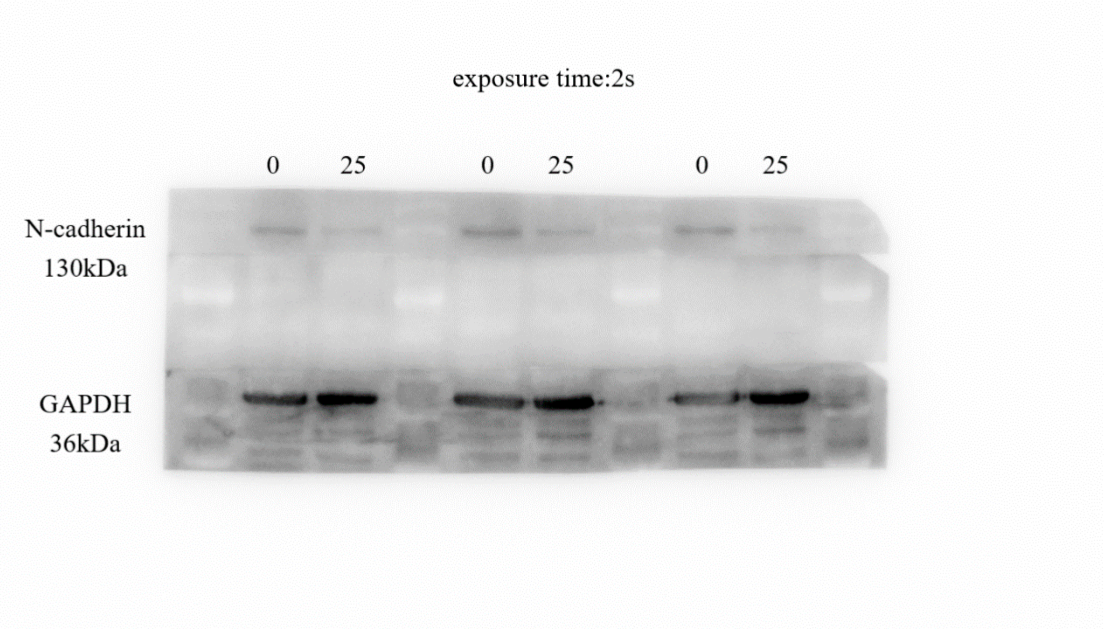

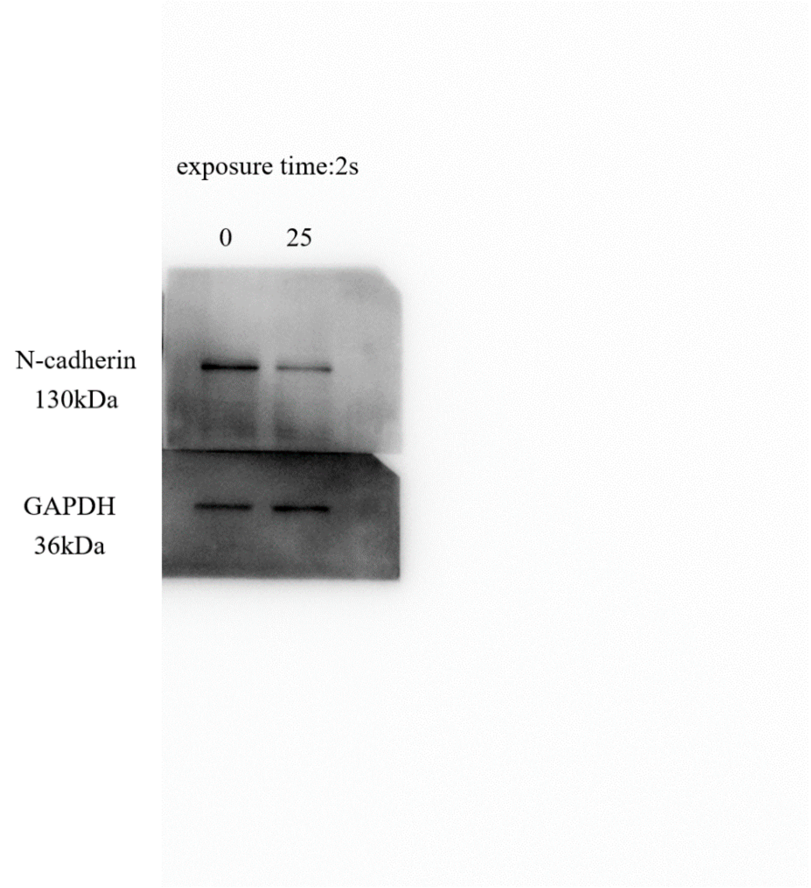

Suppl. Figure S3. Protein expression of N-cadherin.

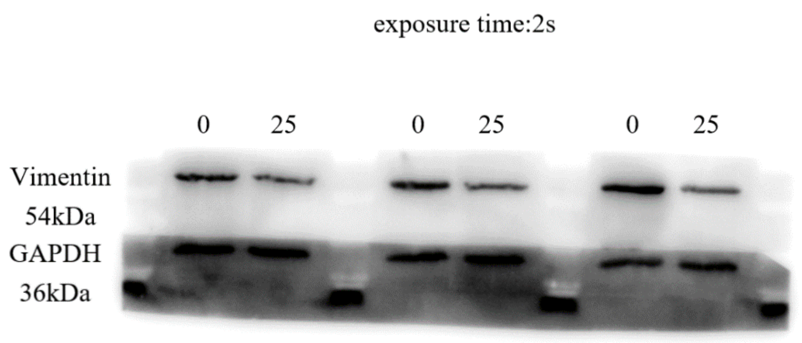

Suppl. Figure S4. Protein expression of Vimentin.

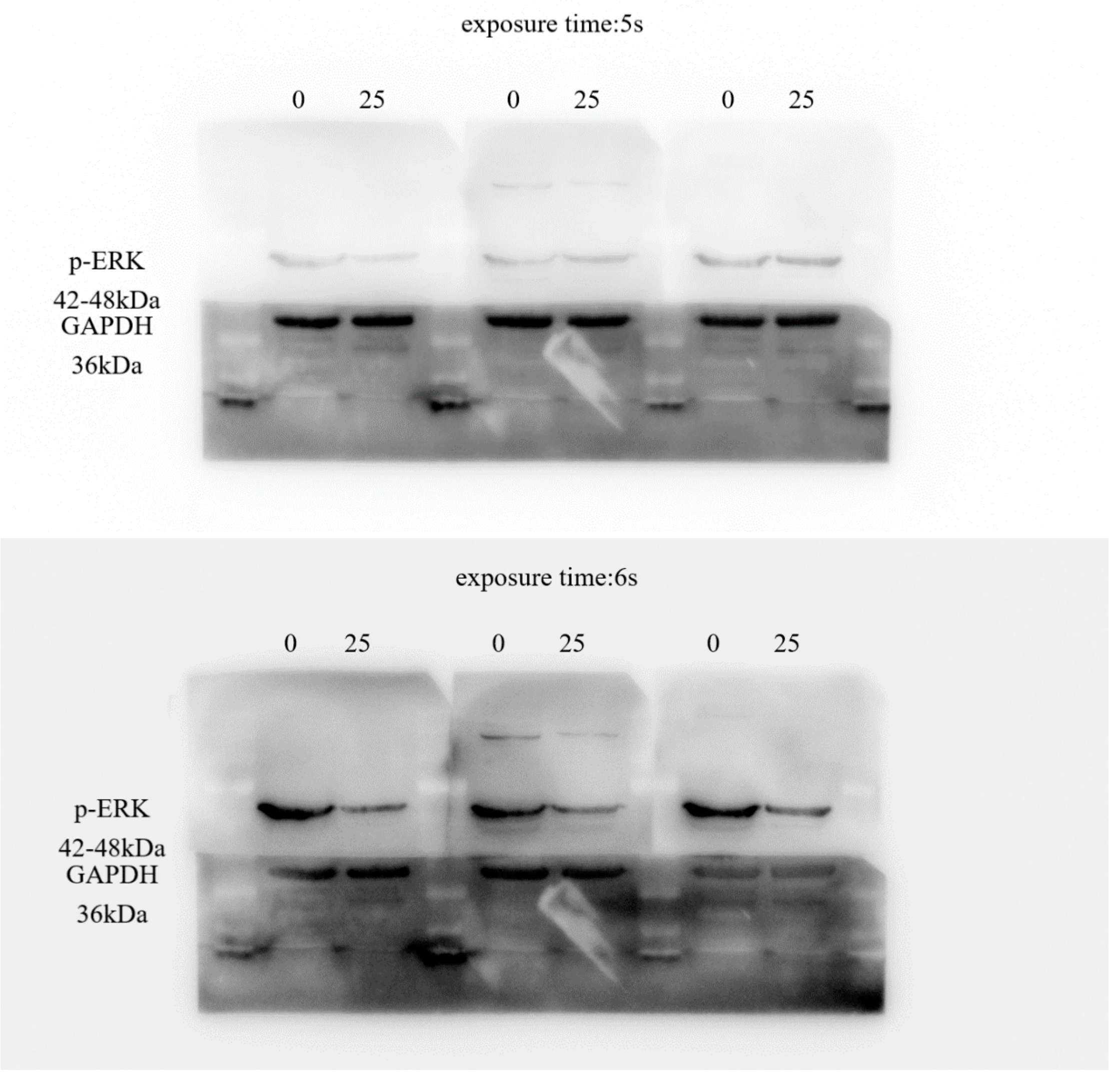

Suppl. Figure S5. Protein expression of p-ERK.

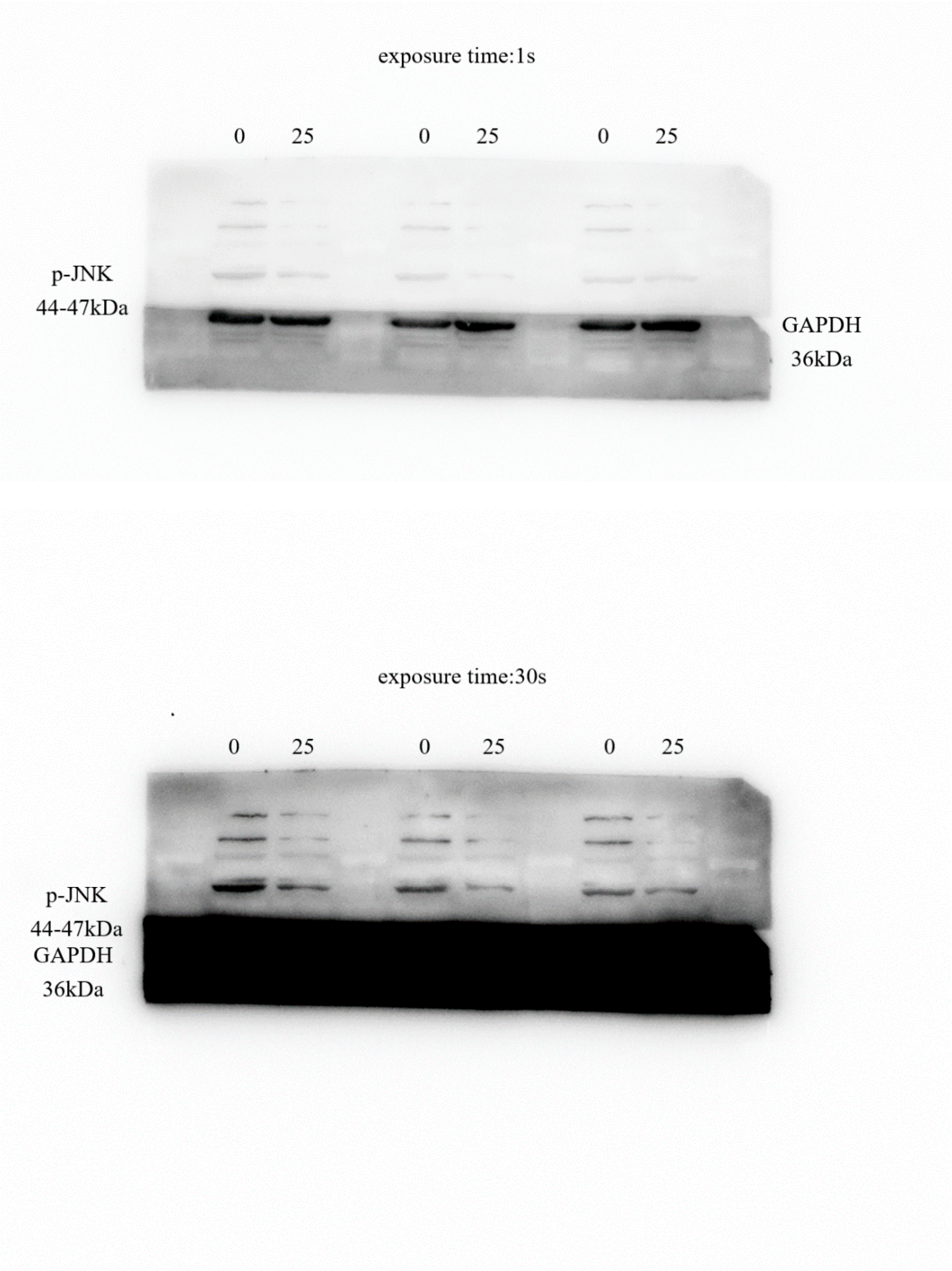

Suppl. Figure S6. Protein expression of p-JNK.

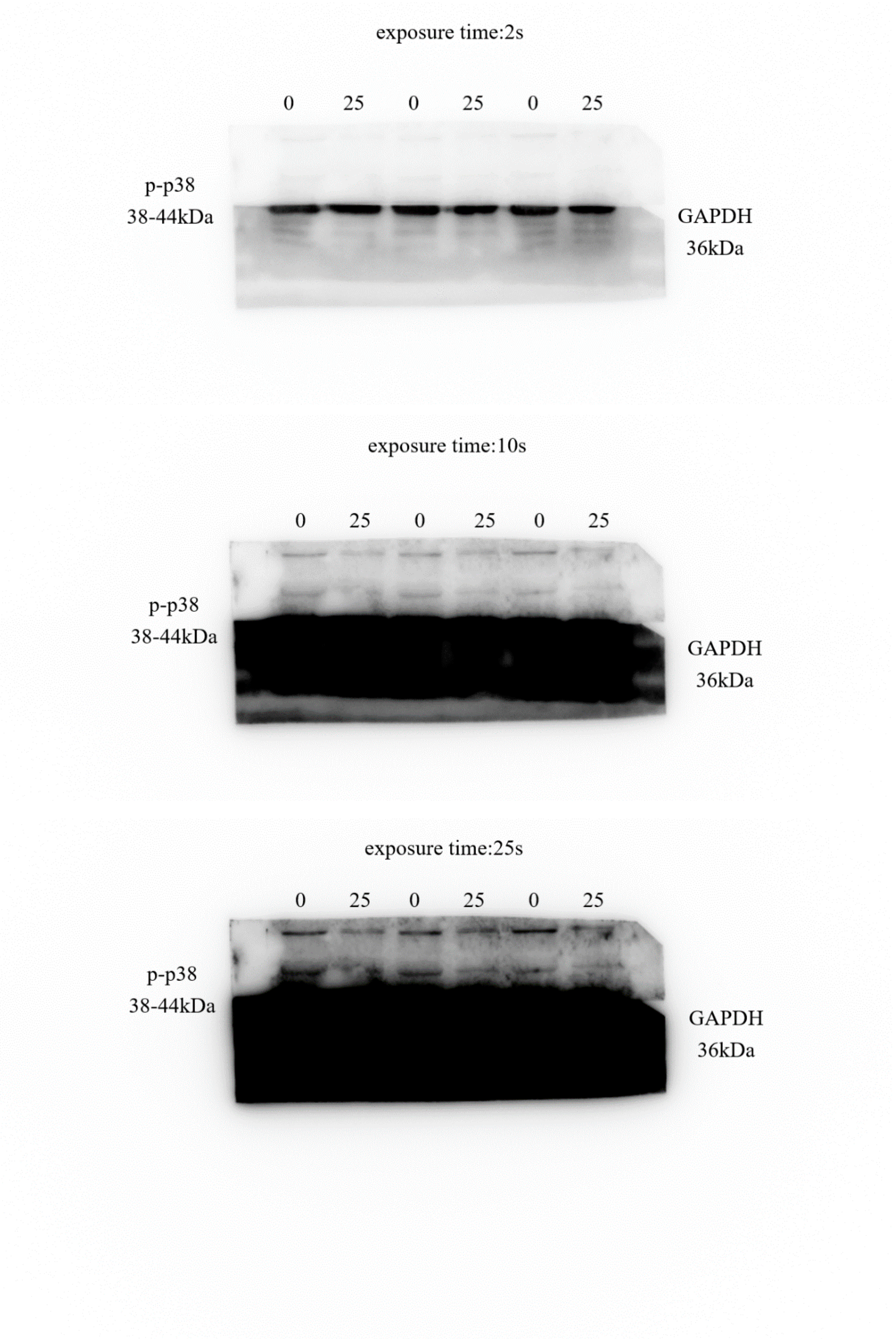

Suppl. Figure S7. Protein expression of p-p38.

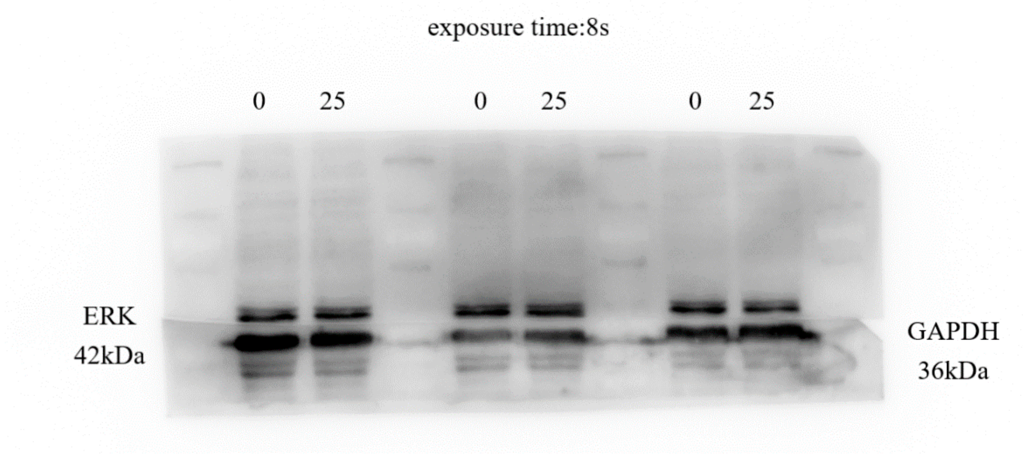

Suppl. Figure S8. Protein expression of ERK.

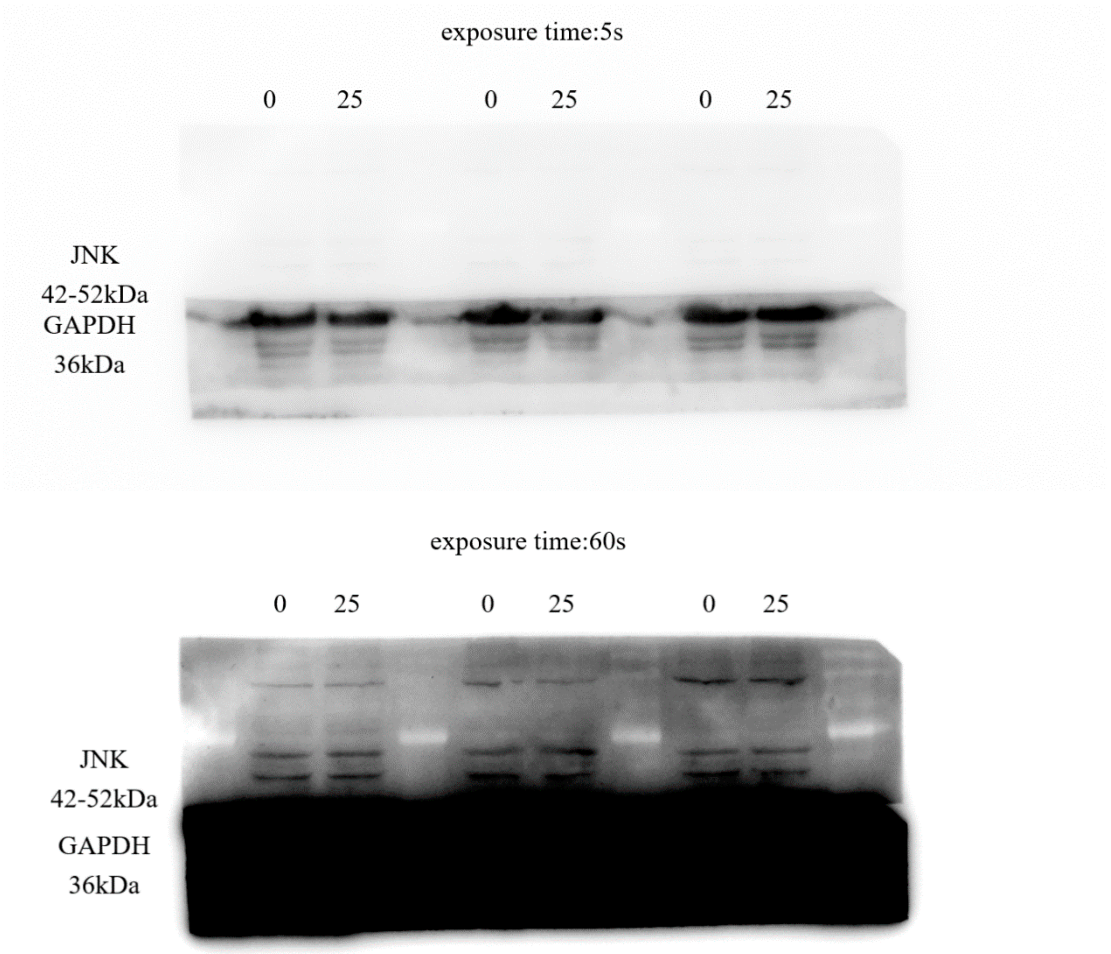

Suppl. Figure S9. Protein expression of JNK.

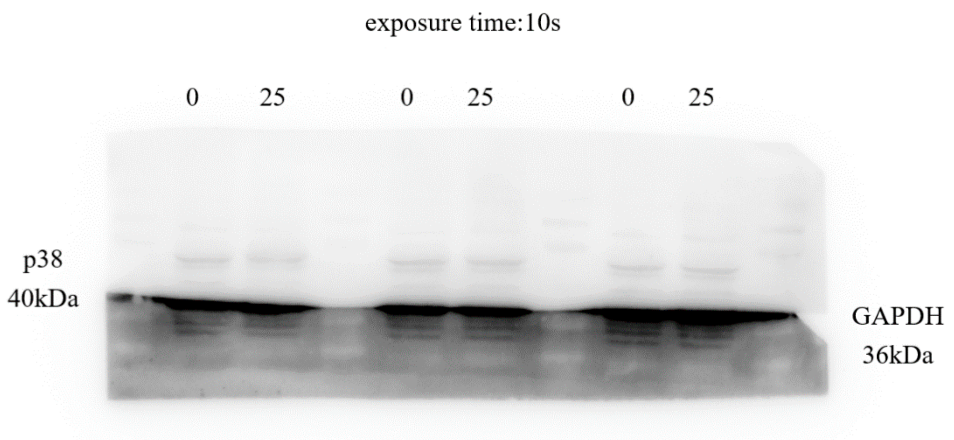

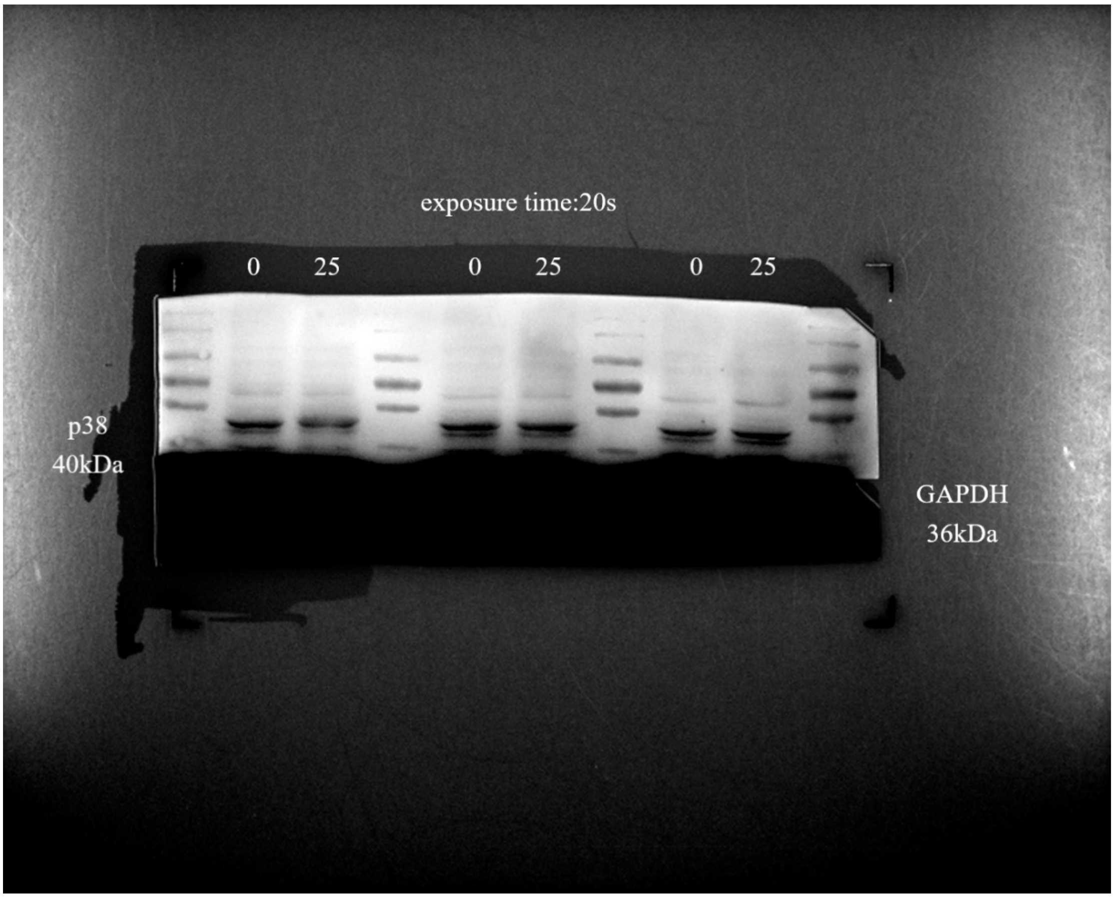

Suppl. Figure S10. Protein expression of p-38.
